# The sufficiency-based, the de-growth, and the official – Critical remarks of the electricity chapter of the Hungarian national energy and climate plan based on a comparison to alternative scenarios

**DOI:** 10.1016/j.heliyon.2024.e39863

**Published:** 2024-10-26

**Authors:** Béla Munkácsy, Csaba Csontos, Ádám Harmat, José Campos

**Affiliations:** Eötvös Loránd University, Faculty of Science, Department of Environmental and Landscape Geography. Budapest, Hungary. H-1117, Budapest, Pázmány Peter Sétány 1/A, Hungary

**Keywords:** Sufficiency, De-growth economy, Strong sustainability, Energy dependence, Electricity sector, EnergyPLAN

## Abstract

Hungary relies heavily on fossil and nuclear fuel imports from Russia. An urgent challenge is to develop a more sustainable and more independent energy system. There is a 2019 and a 2023 (draft) version of the National Energy and Climate Plan (NECP) but they arguably do not follow this direction. However, there are also alternative scenarios for Hungary that propose more comprehensive changes. This paper compares the targets proposed by the Hungarian NECPs to those proposed in two alternative studies: the sufficiency-based European-focused CLEVER (Collaborative Low Energy Vision for the European Region) scenario (created by a research team including 26 partner organisations from 20 European countries), and the de-growth-based “This Way Ahead” (TWA) scenario (which is the result of a Hungarian inter-university research, coordinated by the ELTE University's energy geography research group). A new element of this recent study is the simulation of hourly electricity supply and demand in these scenarios using EnergyPLAN to compare import requirements, potential surplus generation, and self-sufficiency. The review shows that the NECPs need to be reconsidered to increase the share of decentralized renewable electricity supply in a way that allows progress without increasing energy use and environmental burdens. Priority must be given to the rapid expansion of wind energy capacity by at least 10–20 times, which should be complemented by biogas-based flexible energy supply.

## Highlights

• Environmental sustainability needs to be an integral part of NECPs.
• Alternative scenarios could be the reference for incorporating energy sufficiency.
• EnergyPLAN analysis suggests the potential to reduce dependency.

## Abbreviations:

CCGT -: Combined-Cycle Gas Turbine
CLEVER -: “Collaborative Low Energy Vision for the European Region” scenario (published in 2023)
CSO -: Hungarian Central Statistical Office
DSM –: Demand-side management
EU –: European Union
EnergyPLAN –: energy modelling tool
GIS –: Geographical Information System
HFC –: Hydrogen Fuel Cell Vehicles
LTS –: Long Term Strategy
MS –: Member States (of the European Union)
NECP –: National Energy and Climate Plan, Hungary (published in 2019)
NGO –: Non-Governmental Organisation
PP –: Power Plants
RE –: Renewable Energy
TWA –: This Way Ahead scenario (published in 2011)
TSO –: Transmission System Operator
WAM –: “With Additional Measures” scenario of NECPs
GHG –: Greenhouse gas emissions

## 1 Introduction

Achieving the targets proposed in the EU's Fit 55 and later in the REPowerEU, requires Member States (MS) to move away from Russian energy sources by significantly reducing the reliance on imports before 2030 [1]. At the same time, it is necessary to shift from coal and from fossil fuels in general, to clean energy sources with a focus on social justice based on the EU's Just Transition Mechanism [2].

Hungary relies on imports to meet 87 % of its energy needs [3]. More alarmingly, the dependence is overwhelmingly connected to one single country, Russia. Before the Russia-Ukraine war, more than 90 % of oil and natural gas imports and 100 % of the nuclear fuel came from Russia [3]. As of mid-2024, no significant progress has been made in this area [4], the need remains for a complete transformation of the energy supplies by increasing the cooperation with neighbouring countries and ultimately, to develop a sustainable and much more independent energy system, based on local resources and lower primary energy demand.

A multi-disciplinary energy planning and energy policy that takes environmental and resource management aspects into account should be the basis for the transition to a sustainable energy system. This paper compares the objectives of the Hungarian government's NECP 2019 [5] and NECP 2023 [6] with the concepts proposed in two alternative energy planning studies, namely the “This Way Ahead” (TWA) scenario [7] and the “Collaborative Low Energy Vision for the European Region” (CLEVER) scenario [8]. All four scenarios have already been published and the results are freely available. The authors of this study have been involved in the development of the latter two documents.

The aim of the paper is to highlight suitable targets (from the sustainable energy planning perspective) for the development of the energy system by comparing four scenarios. With the comparison, the paper brings to attention the concepts used in the Hungarian NECP. The targets compared are the final energy use, electricity use, development of renewable energy sources and reliance on energy imports. The paper contributes a methodology composed of a qualitative evaluation of general targets and a simulation-based quantitative analysis of renewable electricity targets to compare the different energy scenarios. The research is significant because it summarizes the targets and reveals which development plans exploit the existing potential of renewable energy, and which plans lack urgency in the energy transition. The paper can facilitate the work of researchers and Hungarian decision-makers interested in reducing energy dependency and increasing sustainable energy use.

The paper is structured as follows. Section 2 summarizes a studies comparing energy scenarios. Section 2 defines the concept of sufficiency in energy planning. Section 3 describes the methodology. Sections 4 is the quantitative comparison of the energy targets. The implication of the different targets is presented in Section 5. The key findings and recommendations are listed in Section 6.

## 2 Literature review

### 2.1 Research focusing on the comparison of energy scenarios

Energy scenario comparison studies consist of (i) a correlation of the technical aspects of models (such as which types of power stations are considered in the scenario, utilised energy sources, and storage options) and/or (ii) a comparison of the results (such as the greenhouse gas emission, energy use, and share of renewable energy, ratio of imports) [9]. In both groups, studies can use the quantitative (such as the capacity of power stations, amount of energy use, and share of imports) and the qualitative approach (such as social and environmental consequences of choosing a certain energy source). In the quantitative approach, a set of indicators are used for evaluating the results. In the qualitative approach typically the theoretical characteristics of each model or scenario are compared.

This paper compares model results by identifying the similarities and differences between the results (such as electricity use, the share of renewables, and the choice of energy sources). The models compared differ in many aspects, with significant differences even between the old and new Hungarian NECP scenarios, for example, population change and the extent of renewable energy use.

The analysis published in Limits to growth is a classic example of such scenario comparisons. Twelve very different models have been explored in that research on the global resource management system context [10]. The question was what systemic changes would lead to sustainable scenarios. The present research has a similar aim, in that it seeks to answer the question of whether the current official strategy could show the proper direction to a sustainable and less dependent energy system and whether the sufficiency principle could be applied to put Hungary's energy management on a sustainable path in the long term.

For such, the models compared in this study also used very different assumptions and approaches. The NECP WAM and WEM scenarios are based on the HU-TIMES model, that optimize the energy system on a least-cost basis, also using exogenous variables. These includes, besides the GHG-reduction, energy efficiency and renewable energy targets, also planned capacities which are based broader political decisions, like new nuclear power capacities and CCGT plans. On the other hand, the modelling approach of CLEVER and “This way ahead” (both MS Excel-based models) exclude the future pipeline projects and optimize the outcomes to reach more ambitious renewable energy, energy efficiency and GHG-reduction targets from a combined technical-environmental and social point of view. While the different approaches do not allow to compare the optimization method of the models, the outcome results are comparable, as the modelled energy system is the same. The aim of the analysis is precisely to draw conclusions on the basis of these differences regarding the realisation of the energy transition, for example, the steps that need to be taken.

Regarding the literature on energy scenario comparison, one example is the work of Lunz et al. [11]. In the case of Germany and its “energiewende” (energy transition), the government has been investing heavily in energy planning research, as Lunz et al. [11] have been able to compare 62 potential future electricity systems for Germany for the year 2050. Their methodology consisted of analysing scenarios by calculating the residual load with an hourly resolution for one year, considering demand and electricity generation from wind and solar PV. The study did not highlight one electricity system as the optimal solution but proposed a solution space identifying advantages and disadvantages. Another example is the work of Naegler et al. [12] who compared 26 scenarios. The analysis shows that there are many possibilities of what a climate-neutral energy system in Germany could look like in 2050. There is surprisingly little consensus on even the most basic issues, such as final energy demand, which reflects the high degree of uncertainty inherent in modelling long-term scenarios. According to the paper, a near- or fully carbon-neutral energy system will rely heavily on new, partly immature technologies. As their further development - in terms of applicability, efficiency, and cost - is difficult to predict, their future deployment is highly subject to various assumptions made by modellers. This explains the wide variety of solutions presented in the studies.

In a similar exercise, Xexakis et al. [13] compared 82 electricity supply scenarios for Switzerland for the year 2035. The study showed that 72 of these scenarios rely on fossil fuels or net imports. The study also consulted stakeholders, about their perspectives of the scenarios (for example, preference for 100 % domestic renewable energy). The study revealed that most stakeholders preferred a 100 % renewable electricity supply by 2035.

There are several critical analyses of NECP scenarios in the scientific literature, as well. Williges et al. [14] proposed a framework to assess economic effects, climate effectiveness, and social aspects in the NECPs. They carried out case studies for Austria, Greece, and the Netherlands. Their results indicated that the contribution to GHG reduction targets may be overestimated because life-cycle analysis is often missing. Stamopoulos et al. [15] investigated the economic impacts of the Greek NECP, particularly the impact of investing in renewable energy. Their results show that even though lignite has a significant role in the Greek economy, developing renewables brings the opportunity for value-added and the creation of jobs.

Thimet and Mavromatidis [16] highlight the challenges caused by the considerable number of different studies and results, as policymakers cannot navigate the results of multiple model-based studies and translate the scenarios presented into policy recommendations that they can act on. This also highlights the importance of summarizing scenario results for a given region.

As for the resent research topic, there are just a few comprehensive energy scenarios for Hungary, such as TWA and CLEVER. One of them is developed by the Wuppertal Institute and Energiaklub (a Hungarian NGO) [17], which is comprehensive and contains a sustainable (called Green) scenario with a time horizon of 2050. Having the year 2010 as the reference, the results include information about the costs and long-term impacts of different energy choices highlighting potential benefits, such as a 70 % decrease of natural gas utilization and supply of up to 83 % of Hungarian electricity from RES by 2050. Another comprehensive analysis by Sáfián [18] outlines scenarios of the Hungarian energy system that point towards a 100 % renewable energy future. The EnergyPLAN software was used to create two alternative models to investigate the energy system of 2009 that could have been operated in an optimised way from the environmental point of view, within the existing infrastructure, and reducing carbon dioxide emission by 10 % [18].

### 2.2 The concept of sufficiency and degrowth in the energy planning literature

The concepts of sufficiency and de-growth are often overlooked in energy transition research and official policy papers [19]. Energy sufficiency refers to strategies for a technical, systemic, and cultural transformation toward sustainable restriction of energy demand [20]. Degrowth can be described as the planned reduction of energy use and resource depletion [[21], [22], [23]]. The cultural transformation aspect is of critical importance because the combination of knowledge and ethical elements could be a motivation behind energy-saving actions [24].

There is a qualitative (social science-related) and a quantitative (technical) approach to sufficiency. In a more qualitative approach, sufficiency means that a need is satisfied [25,26]. The second, more quantitative approach specifies a clear target and is more objective by defining points of reference [27]. One example of the quantitative approach is the level of per capita energy use beyond which there is little to no associated increase in quality of life [28]. The range of energy sufficiency values is estimated to be 60–221 GJ per capita per year. The large variation is mainly explained by climatic differences, especially significant differences in heating energy demand. Another example is the Policy Brief of Odyssee-Mure project [29] which describes some of the possibilities to measure the energy sufficiency level of different countries. In the Odyssee Database, there are energy sufficiency indicators for households (as average floor area per person), transport (as car travel mileage per person), and the service sector (as floor area per employee in offices).

While the term degrowth may seem negative to some, Hickel [21] and Sugiyama et al. [30] argue that the concept does not mean the reduction of quality of life, but instead, an improvement of human well-being that is not dependent on economic growth. Kallis [31] argues that applying the concept of no-growth will allow us to build a society that lives better by using less energy and fewer raw materials. Regarding some recent challenges, Keyber and Lenzen [32] suggested that energy planning research should thoroughly consider degrowth scenarios because they can contribute to climate targets and overall sustainability. The Zero Carbon Britain project [33] offers one of the first and one of the most thoroughly developed concepts for multidisciplinary energy planning with growth-reducing elements. This can be explained by the fact that, in addition to the usual technical aspects, social aspects, such as the consumer's behaviour, are considered. The project, which has been running for more than 15 years, has published several documents to support its practical implementation [34].

The Danish Society of Engineers published two alternative scenarios [35,36] to show the way to 100 % renewable energy systems, which suggest that it is possible to continue economic growth while implementing climate mitigation strategies.

## 3 Methodology

The basic idea of the research is to compare the electricity-related chapters and basic objectives of four energy scenarios previously published. The reason for this focus is that electrification is expected to be pervasive in all sectors of the economy, which is widely believed to lead to an increase in electricity supply and demand [37]. However, the energy system of today is very inefficient along the entire energy chain, therefore efficiency needs to be significantly improved, and the energy mix needs to be transformed radically to reduce its environmental burden. There are also significant differences among the scenarios from an environmental (including climate protection) and social point of view.

### 3.1 The focus on the electricity sector

While the assumptions vary significantly among the four scenarios, the input data and level of resolution are similar. The following indicators were used for the scenario comparison.

• C1 Projected total energy use.
• C2 Projected electricity use.
• C3 Projected electricity supply.
  o C3.1 The importance of each renewable energy source in the scenario.
  o C3.2 Developments in the efficiency and capacity factors of the technology.

C1 and C2 are connected to the sufficiency concept, as well as to other important elements, such as the number of consumers. This may also shed light on the extent to which energy efficiency improvements were expected in each scenario. C3 helps visualize the different approaches to environmental sustainability and energy independence goals. C3.1 describes the characteristics of electricity generation but does not describe the environmental impact of site selection and equipment use. C3.2 is relevant because an improvement in the capacity factor will affect the achievability of the visions.

### 3.2 Description of the published scenarios that are used in the comparison

This section describes the context in which the energy scenarios were created, and the approach used in each of them. It should be highlighted that the scenarios were previously developed by other researchers and not all assumptions are described in the present paper. Table 1 is a summary of the main characteristics of each scenario.

**Table 1:** Overview of the main characteristics and implications of the scenarios. The table was compiled based on [[5], [6], [7], [8]].

	This Way Ahead (TWA)	NECP 2019 WAM	NECP 2023 WAM	CLEVER
Environmental pillar	high	low	low	medium
Social pillar	high	medium	medium	high
Economic pillar	medium	high	high	medium
Implications (scale of required systematic changes)	high	low	low	medium

As far as the main characteristics of the scenarios are concerned, it is useful to present the main differences in terms of the three pillars of sustainability. It is also important to note that this inevitably leads to further simplifications, but it does illustrate properly the differences in the approach. For the environmental pillar, this is simplified to the extent of climate mitigation, for the economic pillar, to the extent of final energy use. The social pillar is the most challenging, as it is difficult to find such aspects in energy scenarios, so in this case Table 1 focused on well-being related to the projected environmental condition. As for the implications, we consider the extent of the expected changes in the socio-economic system as an appropriate basis for comparison.

It is also important to stress that in the case of the CLEVER scenario, only the calculations and findings for Hungary were taken into account. This means that the picture that emerges here is less ambitious than if the entire CLEVER geographical area (EU27 + Norway, Switzerland and UK) was considered since CLEVER is much more ambitious for the more economically developed countries of the research area.

#### 3.2.1 Description of the “This Way Ahead” energy scenario

The software-based “This Way Ahead” (TWA) degrowth scenario (available at [6]) was developed in 2010–2011 in cooperation between researchers of ELTE University (Budapest, Hungary), the Environmental Planning and Education Network (a Hungarian NGO) and the International Network for Sustainable Energy - Europe (INFORSE-Europe, an international NGO).

TWA applies the concept of degrowth [38] by assuming a relatively stable economic production until 2050. According to its concept, in the first decades of the energy transition, when the new system needs to be established, the economy should grow to create the new infrastructure. Afterward, the production in the whole economy will be stable or will decrease by enforcing the principles of strong sustainability [39]. This approach can be explained by the fact that ecological footprint and biocapacity analyses show that Hungary is also experiencing an elevated level of overshooting [40], which needs to be reduced by curbing overconsumption.

The analysis used the INFORSE software [41] to look 40 years ahead in the energy sector in 5-year steps. The research aimed to investigate whether the transition to a sustainable energy system is feasible in Hungary and in what timeframe. In terms of methodology, it pioneered in Hungary the use of GIS methodology to map the RE potential of certain RE sources, such as wind energy. Estimates of the potential for efficiency and sufficiency were made through a literature search. The results showed that a full transition to 100 % renewable energy would have taken 30 years from 2010. This would have required a significant reduction in the country's energy use through increased efficiency and awareness. The analysis shows that reductions could be achieved not only in primary energy use but also in electricity use, despite the electrification of transport and heating. The concept and the results were published in two volumes (in 2011 and 2014), only in Hungarian.

#### 3.2.2 Description of the NECP WAM scenarios

As a part of the EU's energy and climate strategy, each MS had to develop a National Energy and Climate Plan (NECP) for the period 2021–2030. NECPs describe, among other things, how the MS plans to address GHG emission reduction, energy efficiency, RE capacities, interconnections, and research/innovation. The latest complete version of Hungarian NECP was published in 2019 (available at [5]). The NECPs have a more progressive scenario called “with additional measures” (WAM). The updated draft version was published in September 2023 (available at [6]). In this paper, these documents will be referred to as “NECP 2019 WAM” and “NECP 2023 WAM”.

The NECP figures are based on modelling, using the HU-TIMES model. The TIMES model - previously known as MARKAL [42] - has been developed by the IEA (International Energy Agency) since 1978, and it is based on least-cost optimization. Its application in Hungary was developed to establish the NECP. It covers the entire Hungarian energy sector. The model itself is neither open source nor available to the public, therefore it was not possible to use it for this modelling exercise.

The four key objectives of the NECP 2019: a) to promote energy sovereignty; b) to foster energy security; c) to maintain the results of the “utility cost reduction” program; d) to decarbonise energy supply. The NECP 2023 has similar goals. However, the new nuclear reactors at the core of these concepts, which are planned to require Russian technology and Russian fuel imports, are in no way a step towards energy sovereignty. Against this background, energy security is also questionable. As for the utility cost reduction program of the government (which started in 2014), it has serious negative effects on energy efficiency, sufficiency, and decarbonisation [43]. The government's approach prioritises economic growth and neglects climate protection. This is illustrated by the following scenario's statement. “Reducing energy is naturally a priority, but in the case of economic growth, neither industry nor transport can be constrained in their use of energy” [5].

Finally, without ambitious renewable energy targets (27 % RE ratio in final energy use by 2030 in NECP 2023 WAM), decarbonisation in all sectors, but especially in electricity generation, will be called into question.

#### 3.2.3 Description of the CLEVER energy scenario

CLEVER proposes a decarbonisation pathway for the EU through energy efficiency, sufficiency, and renewable energy development (fully available at [8]). The scenario applies a bottom-up approach. National scenarios are aggregated to reach carbon neutrality and a 100 % renewable mix at the European level by 2050.

In the context of the study, sufficiency means changing society's resource-intensive activities to be compatible with Earth's capacity without compromising well-being. As for the elements of sustainability, it can be stated that the social part has a key role, together with the environmental component.

The scenario suggests a range of energy use that all MS should achieve for a good quality of life. However, in some MS the per capita energy use may increase to reach this overall European average, which is a contradiction to Ehrlich and Holdren equation [44] which suggests that reducing the per capita energy use contributes to reducing environmental pressures. Nevertheless, there is an understanding that sufficiency requires changes in current general behaviours, social practices, and norms, as well as in the organisation of society, e.g., spatial planning and the prevailing socio-economic paradigm [45].

Its methodology is composed of three steps. In the first the energy demand in each sector (buildings, transport, etc.) is estimated incorporating the sufficiency concept. The second consists of applying improvement in efficiency to the estimated energy demand and choosing energy carriers according to planetary limits [46]. The last step is the calculation of energy supply needs from renewable sources. Using the ideas mentioned above, the CLEVER project has recently produced a comprehensive scenario for MS and two additional countries, demonstrating that there is a theoretical potential for a shift to RE across the region by applying the tools of sufficiency and strengthening cooperation between countries [[8], [47]].

It is important to note that CLEVER's ambitious targets imply a real change of direction mainly for richer countries, while for poorer countries (such as Hungary), the scenario implies an increase in energy use. In the present study, we have only looked at the vision for Hungary.

### 3.3 Model of electricity import, surplus generation, and self-sufficiency in the four scenarios

The EnergyPLAN tool [37] was used for modelling the electricity demand and generation capacity described in each scenario. This freeware tool was chosen because it was specifically designed for simulating exogenously defined energy systems, particularly systems with high shares of renewable sources. Moreover, this tool allows to compare totally different strategies and facilitate the discussion of suitable development pathways for the energy system. By demonstrating the consequences of different concepts, a preferred development pathway can be selected [48] based on given criteria, such as independence from external resources. A detailed description of EnergyPLAN and its guiding principles was presented by Lund et al. [37].

Previous studies have applied EnergyPLAN to model the Hungarian electricity system [18,49], however, the present paper is the first to use the tool to model a nearly 100 % renewable-based electricity scenario. The purpose of our analysis was (i) to estimate electricity import requirements in one year (TWh/year) due to insufficient generation of the portfolio and (ii) to estimate the theoretical surplus generation due to non-flexible and intermittent generation capacity. This information can facilitate the choice of a development pathway that comprehends energy independence and decarbonisation.

The model was developed because the estimation of import requirements and surplus electricity in the TWA and CLEVER were not based on hourly simulations and the model of the NECPs with hourly resolution is not available. The EnergyPLAN model does not consider any other assumptions other than those described in each published scenarios; all inputs are exogenously defined and are summarised in Table.

The simulations considered the electricity demand and generation of the years 2030, 2040, and 2050 (where applicable). Additional input was the historical hourly distribution of electricity demand using 2022 as the reference year obtained from the Hungarian Transmission System Operator (TSO) [50]. Each scenario projects its own total yearly electricity demand (in TWh). The historical data on the hourly distribution of 2022 (available from Ref. [50]) was used in all scenarios, but the total yearly demand was adopted according to each scenario (as this parameter can be easily modified in EnergyPLAN). Potential modifications to the hourly distribution of electricity demand are not specified in the originally published scenarios. Three years (2017, 2019, and 2021) of hourly distribution of electricity supply from wind, solar, and hydropower were also obtained from the TSO. These years were chosen because of the quality of the data available as other years have missing data points.

To facilitate the reproducibility, the parameters of the EnergyPLAN model are displayed in Table 2. By inputting these values in the demand branch and supply branch, one can obtain the import requirements and surplus electricity results. In the EnergyPLAN nomenclature, these refer to “Import Electricity” and “Export Electricity” in the results sheet. The electricity demand should be placed in the demand branch while the thermal power station capacity should be placed in the supply branch (central power supply) under the “Condensing PP2” tab. The nuclear power station capacity should be placed in the central power supply branch. The “Variable Renewable Electricity” branch should be used for adding the capacity of wind turbines, photovoltaics, and hydropower. The remaining files to reproduce the simulation (historical data on renewable energy output) will be made available upon request.

**Table 2:** Parameters for the simulation of each scenario in the EnergyPLAN tool.

	EnergyPLAN parameter					
	Electricity demand (TWh)	Thermal power stations (MW) - “Condensing PP2” tab in EnergyPLAN	Nuclear power station (MW)	Wind turbines (MW)	Photovoltaics (MW)	Hydropower (MW)
Scenario for 2030						
CLEVER	48.90	1050	1850	4146	9847	57
NECP WAM (original 2019)	51.78	1827	4400	293	6454	57
NECP WAM (review 2023)	46.89	3364	4430	1080	12,000	57
TWA	18.78	1697	0	8200	3281	89
Scenario for 2040						
CLEVER	52.46	839	7863	19,432	57	
NECP WAM (original 2019)	59.78	1011	2400	293	11,975	75
NECP WAM (review 2023)	60.23	2395	4430	2000	14,935	57
TWA	14.41	692	0	10,253	5457	95
Scenario for 2050						
CLEVER	60.12	734	0	9000	26,000	57
NECP WAM (review 2023)	82.46	4684	4430	3000	20,365	57
TWA	11.18	222	0	10,253	5457	95

The self-sufficiency in each scenario was calculated by subtracting the hourly generation of domestic resources from the national hourly demand. The following were considered as domestic resources in the calculation: wind power, solar power, hydropower, biomass, and biogas for electricity generation. The following were considered as imported resources in the calculation: nuclear fuel and natural gas for electricity generation. If the result of the difference was zero or a negative value it meant that the self-sufficiency was 100 % in that given hour. If the result was positive, it meant that imports were needed. The yearly value was calculated by the sum of each hour and dividing the result by the yearly demand. Negative hourly values were corrected to zero to calculate the yearly self-sufficiency index. Hourly values of self-sufficiency in electricity generation (%) for one week in January and one week in June are presented in the Chapter 4.

## 4 Results

First, comparisons of total energy use, electricity demand and generation capacities to be built according to each scenario are presented. Then the results of the simulations on electricity imports and surplus generation are presented.

### 4.1 Basic assumptions

TWA assumes that the population remains constant because of the balance between the decreasing number of births and the expected influx of environmental refugees into the area. The population projections considered by the NECP 2019 were carried out by the CSO in 2015 (Table 3) and forecast a significant decline; only 9.17 million people in 2030 and 8.56 million in 2050. The NECP 2023 and the CLEVER are based on the Eurostat projections [51].

**Table 3:** Size of projected population in Hungary (million).

Scenario	2020	2030	2040	2050
TWA	10.000	10.000	10.000	10.000
CLEVER	9.772	9.619	9.441	9.270
NECP 2019 WAM	9.671	9.170	8.865	8.560
NECP 2023 WAM	9.769	9.619	9.441	9.270

Another basic assumption was the forecast of the level of economic development. The NECPs considered that a steady increase in GDP means a proportional increase in total energy use. The TWA considers environmental constraints both in terms of access to resources and assimilation of waste and emissions. The CLEVER does not describe economic development; instead, based on the activity levels described in the scenario, it can be stated that it considers an increase in economic activity in the case of Hungary, but the associated energy use decreases, unlike the NECPs.

### 4.2 Total energy use

**Fig. 1:**
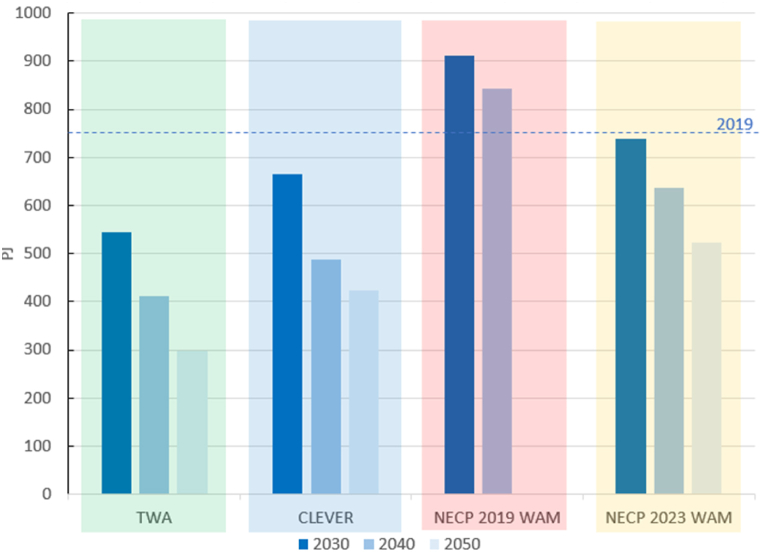
Final energy use of all sectors in 2019 and projections according to each scenario. Note: there is no projection for 2050 in the scenario NECP 2019 WAM.

The final energy use projected in the NECPs is the highest. The CLEVER also projects higher energy use than the TWA but to a lower extent. In 2050 the projection of the CLEVER is 43 % higher than the TWA (Fig. 1). The higher consumption in the NECP 2019 WAM highlights how the DSM measures received less attention in this scenario.

The relatively high final energy use of the CLEVER in comparison to TWA is explained by the vision behind the scenario: the CLEVER does not explore the possibility of reducing energy use to the full extent but rather proposes more easily achievable targets.

### 4.3 Electricity use

**Fig. 2:**
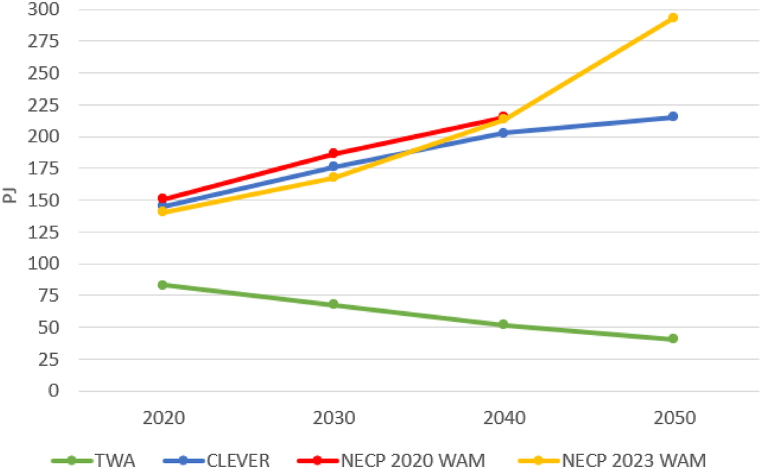
Projected electricity use of the scenarios. The figure for 2020 is actual data in the CLEVER and NECP 2023 WAM, but a projection in the TWA and NECP 2020 WAM which were created earlier. Note: there is no projection for 2050 in the scenario NECP 2019 WAM.

The TWA was developed in 2005 while the other scenarios were developed between 2019 and 2023 which explains the different values in the initial year, 2020, in Fig. 2. In the final year of the TWA, the electricity use is much lower (49 %) than the projected for 2020 because of the assumptions of sufficiency and efficiency of the model. All the other scenarios predict a significant increase in electricity use. From the baseline in 2020, CLEVER projects a growth of 39.2 % by 2040 and 48 % by 2050 in comparison to 2020. The growing tendency is more significant in the projection in the NECP 2019 as there is a 43 % increase already by 2040. This scenario does not cover the period after 2040. The projected growth of electricity use is even greater in the NECP 2023.

**Fig. 3:**
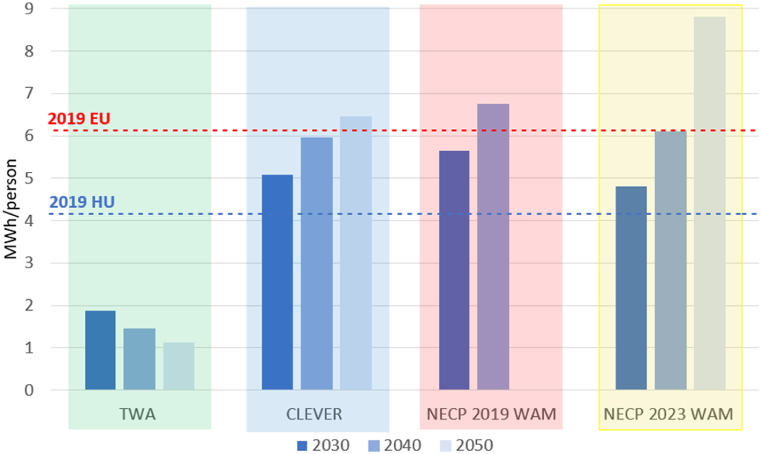
Electricity use per capita (MWh/capita) in 2019 and projections according to each scenario for Hungary and the EU average [52]. Note: there is no projection for 2050 in the scenario NECP 2019 WAM.

A more informative aspect is the per capita yearly electricity use illustrated in Fig. 3, highlighting a significant difference between the four concepts. In 2040, the value of CLEVER is four times higher than the value in the TWA, which can be explained not only by its deep efficiency and sufficiency improvements but also by its no-growth idea. The NECP 2019 WAM is 5 times higher and NECP 2023 WAM is 4 times higher than the TWA. The differences in the projected figures for 2050 are greater.

### 4.4 Electricity supply

**Fig. 4:**
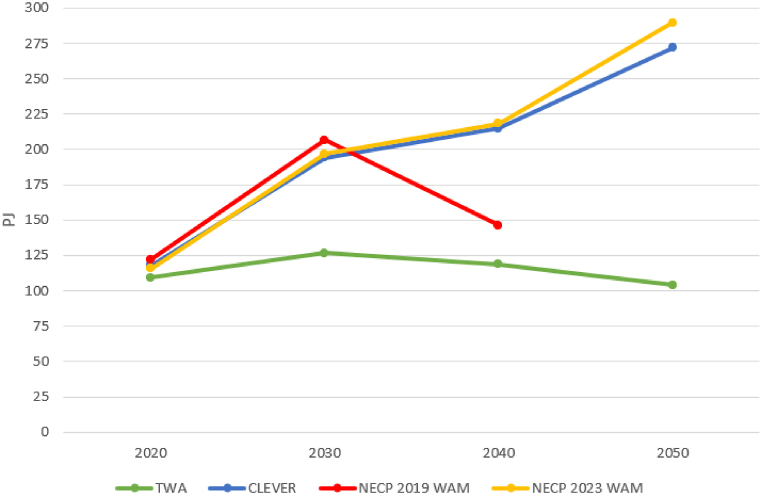
Evolution of electricity supply in the four scenarios. The figure for 2020 is actual data in the CLEVER and NECP 2023 WAM, but a projection in the TWA and NECP 2020 WAM which were created earlier. Note: there is no projection for 2050 in the scenario NECP 2019 WAM.

In the case of the TWA, there is a slight increase and later a decreasing tendency (Fig. 4). In the final year, there is approximately 4 % less supply compared to 2020. Surprisingly, the CLEVER and the NECP 2023 have a similar projection of supply. The NECP 2019 curve is more like the TWA forecast. Considering the increasing environmental pressures associated with increased supply, the question arises as to how far this change in NECP targets is in line with the EU's increasingly stringent approach.

**Fig. 5:**
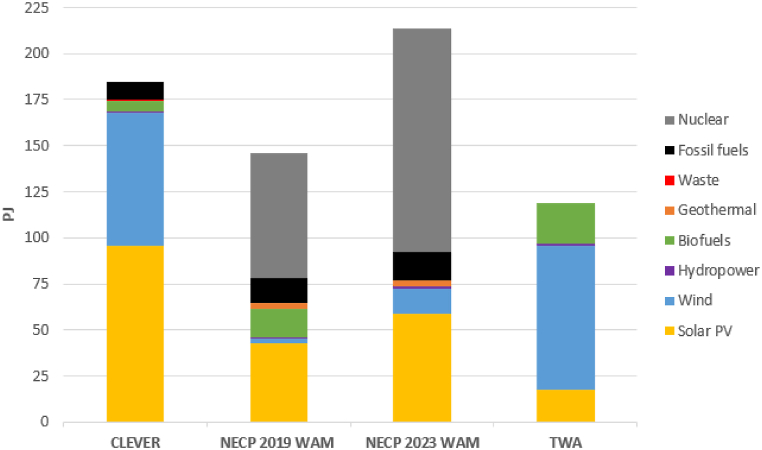
The envisaged mix of sources for electricity supply in 2040.

The envisaged structure and quantity of the electricity supply is presented for the year 2040 in Fig. 5. According to TWA the complete transition to RE would be achievable by this time if its vision had been implemented when the scenario was created in 2010.

Nuclear power is the main type of generation in the NECPs, but it is not part of the mix in the alternative scenarios. Fossil fuel use decreases, however, the NECPs are the least ambitious in this field.

Solar PV, wind power, and biomass are the major renewable sources. However, there are big differences in the use of biofuels in all scenarios and even in the two official scenarios. The NECP 2023 does not contain any significant biomass use. This fact contrasts with the earlier version of the document where the importance of biofuels was larger. Comparing the two alternative scenarios, the ratio of biofuels in TWA is higher because this source is used to produce flexibility on the supply side.

The ratio of wind energy is 75 % in the TWA while it represents 37 % of the total electricity supply in the CLEVER. This technology has limited contribution to the NECPs.

Solar power also accounts for 24 % of total electricity generation in the NECP 2019. It accounts for 52 % in the CLEVER and 28 % in the NECP 2023 in 2040. Its ratio is lower in the TWA.

#### 4.4.1 Renewable electricity generation capacity

**Fig. 6:**
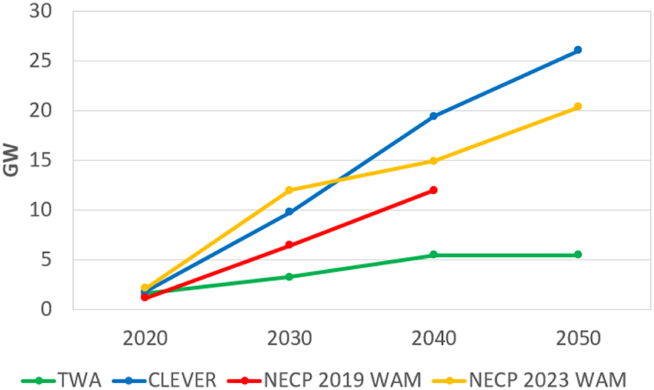
Development of solar PV built-in capacity proposed in the scenarios. Note: there is no projection for 2050 in the scenario NECP 2019 WAM.

The TWA suggests a smaller capacity (Fig. 6) for solar PV than the other scenarios because at the time the study was developed in 2010 this technology was not as mature and cheap as today. Regarding technology development, in Hungary, the yearly average supply was estimated at 125 kWh/m^2^. Nowadays, the supply could be 225 kWh/m^2^. Another reason for the lower value compared to the other scenarios is that green areas are not considered for the installation of solar panels, all the proposed capacity would be installed on building roofs, brownfields, and infrastructure-related areas, for example as noise barriers. Moreover, it was estimated that ∼40 million m^2^ areas would be used (the available area was estimated at 99 million m^2^).

As the CLEVER was recently developed, solar PV has a much larger capacity (more than five times the capacity, as in TWA). Solar PV is the most important technology for electricity generation in the CLEVER.

The proposed solar PV capacity in the NECPs sits between the TWA and CLEVER values, however, this is the most significant RE technology within the NECPs. Moreover, the NECPs’ proposed solar PV capacity is still one of the least ambitious when compared to other EU countries (considering the capacity per unit area).

**Fig. 7:**
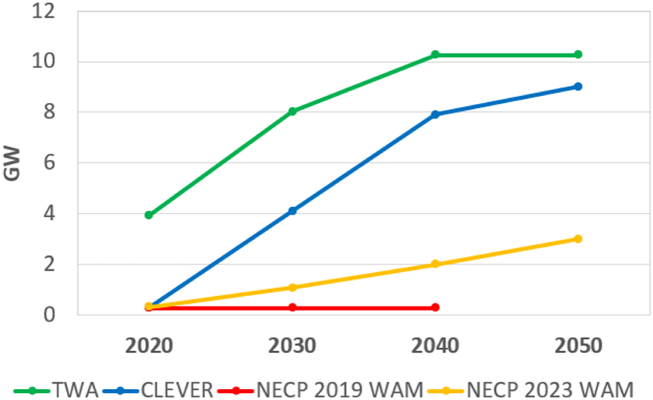
Development of wind energy built-in capacity proposed in the scenarios. Data for TWA in 2020 differs from the other scenarios because this scenario was created in 2010. Note: there is no projection for 2050 in the scenario NECP 2019 WAM.

Wind energy capacity development is presented in Fig. 7. Wind energy is dominant among the RE technologies in the TWA. However, the supply from wind turbines is higher in the CLEVER. The development of wind power technology happened fast in the last decade, but this improvement was not considered in the TWA which is why the supply is relatively lower even though the capacity is higher. By 2050 the CLEVER will have a similar capacity but a higher electricity supply because of a higher capacity factor of the wind turbines.

The TWA concluded that between 6 and 8 % (5600–7400 km^2^) of land was available for wind energy considering the national regulation in 2010. However, due to a change to the regulation in 2016, there is currently no land available for wind turbines, as since then, wind farms cannot be installed within 12 km of built-up areas and areas intended for development. This rule is currently being reconsidered.

According to the NECPs, there will be no significant increase in wind power capacity until 2050, even though the capacity factor of wind turbines in Hungary is around the European average. Moreover, the fleet of wind turbines in the country is old; the latest wind farm was commissioned in 2011.

#### 4.4.2 Developments in renewable energy technology

As previously highlighted, the TWA had conservative assumptions regarding the improvement of the capacity factor of solar PV and wind turbines over the years (Table 4). These assumptions mean that with the same built-in capacity suggested in the scenario, it would be possible to produce more electricity, because of the higher capacity factors of state-of-the-art wind energy technology. The capacity factor values are compatible with historical evolution of the wind turbine fleet and photovoltaics sector in Germany [53] and other regions.

**Table 4:** Capacity Factor values considered in the already published scenarios (%).

	2020	2030	2040	2050
TWA - Solar PV	12.6	12.6	12.6	12.6
TWA - Wind turbines	24.0	24.0	24.0	24.0
CLEVER - Solar PV	15.0	16.0	16.0	16.0
CLEVER - Wind turbines	24.0	26.0	29.0	31.0
NECP 2019 WAM - Solar PV	13.0	11.6	11.3	–
NECP 2019 WAM - Wind turbines	27.0	27.0	27.0	–
NECP 2023 WAM - Solar PV	12.6	12.6	12.5	12.6
NECP 2023 WAM - Wind turbines	25.7	25.7	25.7	25.7

The NECPs do not consider improvements in capacity factors. Interestingly, its 2020 version considers a decrease in the average capacity factor of solar PV technology, according to the forecasted capacity and supply figures. These capacity factors for 2050 for both NECPs are significantly lower than realistically expected.

### 4.5 Electricity imports, surplus generation, and self-sufficiency

**Fig. 8:**
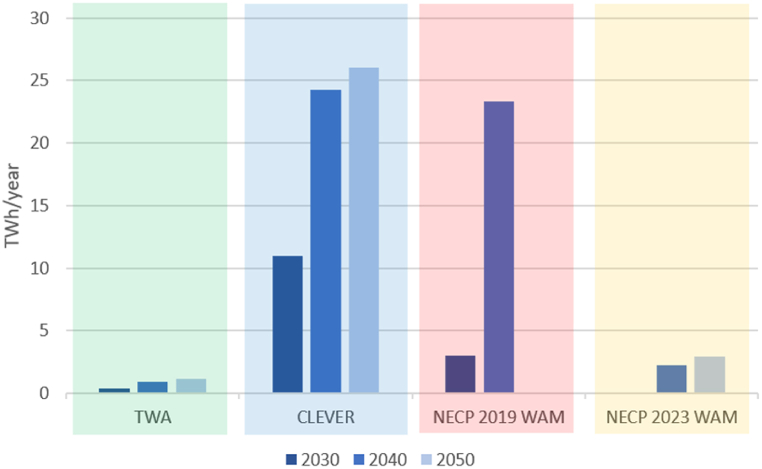
Electricity imports requirement in each scenario. Note: there is no projection for 2050 in the scenario NECP 2019 WAM. Electricity imports requirement in the scenario NECP 2023 WAM in 2030 is nearly zero.

The simulations indicate that TWA requires less electricity imports than any other scenario (Fig. 8). CLEVER requires more electricity imports, however, this scenario contains an increase in electricity use due to the electrification of transportation and heating. Electricity imports in 2030 in the NECP 2019 WAM are between the levels suggested in TWA and CLEVER while the share of renewable electricity is significantly lower. Imports in 2040 increase because part of the nuclear electricity generation capacity will be retired. There is no data for the year 2050 in the NECP 2019 WAM. Interestingly, NECP 2023 WAM requires almost no imports in 2030 because of the enhanced nuclear capacity. After 2030 the imports increase due to higher electricity use.

**Fig. 9:**
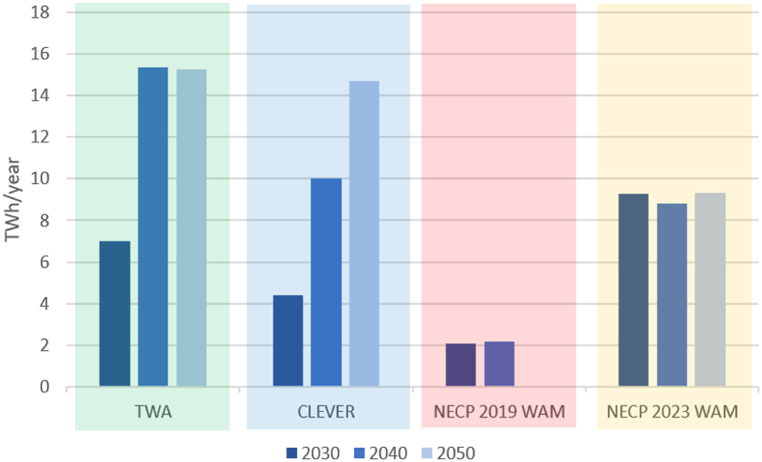
Theoretical surplus electricity generation. Note: there is no projection for 2050 in the scenario NECP 2019 WAM.

The surplus generation (Fig. 9) refers to the output of non-flexible power stations and intermittent generation. In practice, the electricity would not be generated, unless there is the possibility to store or export it.

In the TWA, the large amount of electricity surplus after 2030 is explained by the significant reduction in electricity use. In the CLEVER the surplus increases proportionally to the renewable generation capacity. The NECP 2019 WAM would result in the lowest surplus because the amount of renewable electricity is also the lowest compared to the other scenarios. Despite the increase in renewable electricity, the amount of surplus in the NECP 2023 WAM is relatively constant between 2030 and 2050 because of the large capacity of the nuclear power station.

**Fig. 10:**
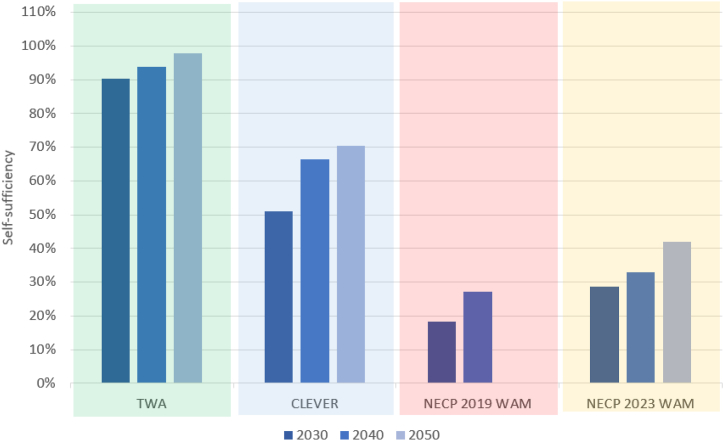
Self-sufficiency in electricity generation (% of the electricity demand that is covered with domestic resources which include wind power, solar power, hydropower, and bioenergy). Note: there is no projection for 2050 in the scenario NECP 2019 WAM.

Fig. 10 highlights the TWA and CLEVER scenarios would result in the highest level of self-sufficiency. This means the share of the yearly national electricity demand that could be covered with electricity generated using domestic resources, namely wind turbines, photovoltaics, hydropower, and bioenergy.

**Fig. 11:**
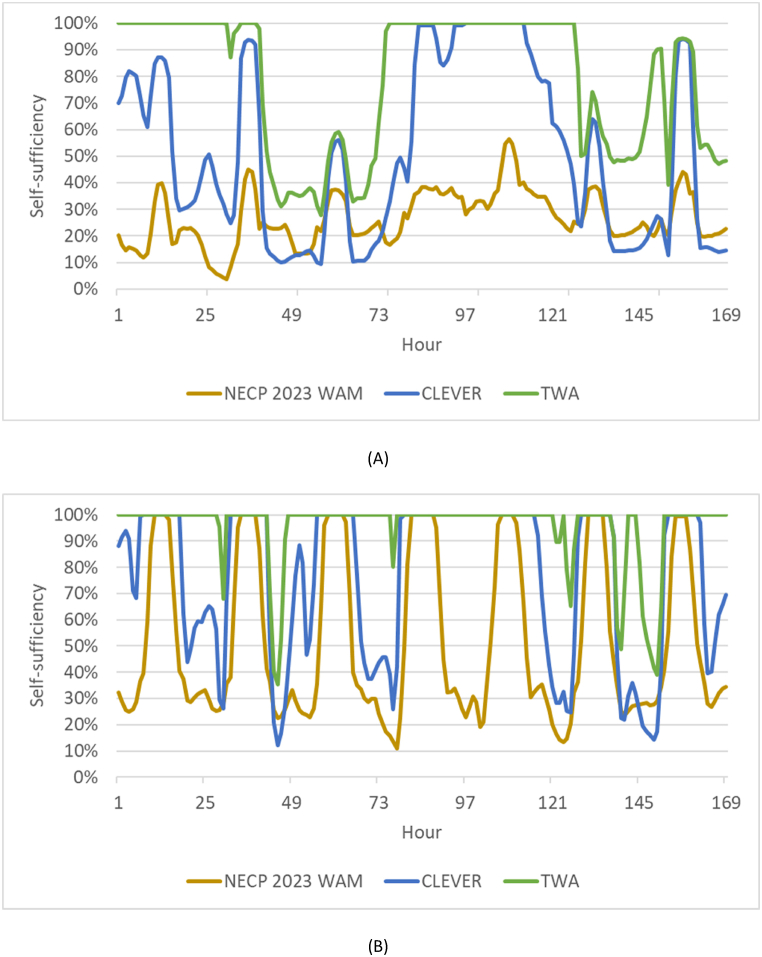
Hourly values of self-sufficiency in electricity generation (% of the electricity demand that is covered with domestic resources which include wind power, solar power, hydropower, and bioenergy). Figure (A) is a representation of a week in January and Figure (B) is a representation of a week in June. Scenarios for 2050.

Fig. 11 shows the hourly level of self-sufficiency (% of the electricity demand that is covered with domestic resources which include wind power, solar power, hydropower, and bioenergy) in one week in January and one week June considering the scenarios for 2050. This interval was chosen because it facilitates the visualization of the hourly results.

## 5 Discussion

### 5.1 Basic assumptions and total energy use

The most significant population reduction was expected in the NECP 2019, which is almost 11.5 %. The decrease is much lower in the NECP 2023 and the CLEVER, 5.14 %. The recent national population policy favours bigger families with tax regulations and subsidies, however, the immigration policy in Hungary is much stricter than the European practice. These are opposing forces on future population numbers. Due to a completely different immigration policy, the TWA assumed a constant population, which is around one and a half million more than the official scenario in 2050.

The size of the population is not the only factor influencing the electricity use. Sufficiency and efficiency measures are the other main influencing factors. The TWA scenario shows that even though there is an assumption of a constant population, electricity use can be reduced when sufficiency and efficiency measures are incorporated. The CLEVER considers a decreasing tendency in the total population but an increase in electricity use. Moreover, the NECPs consider a bigger decrease in population and an even bigger increase in electricity use.

As for the de-growth concept, an almost 30 % reduction in final energy use was possible due to a clear trend in the efficiency of household energy use between 2000 and 2019 [49]**.** In comparison, the Odyssee-Mure project found an improvement of 20 % in overall efficiency between 2000 and 2018 [54].

Contrary to the reduction pathway, the Hungarian government has launched a re-industrialisation programme, under which around 20 major and 20 smaller battery factories are in the pipeline [55] planned to make Hungary the world's second-largest battery producer [56]. This will significantly increase the country's electricity needs, which will be met mainly by buying imported natural gas and building a 1500–1650 MW gas-fired power plant capacity, which is intended as baseload supply [6,57]. This does not seem the right direction for the transition to a more independent, carbon-free, sustainable energy supply.

### 5.2 Electricity use

The de-growth is observed through the electricity use in the TWA scenario. In the TWA there were two elements, the activity level (which is connected to energy sufficiency) and the specific demand (which is connected to the energy efficiency of energy services). The electricity use can be reduced because both the activity level and specific demand should be reduced in most of the sectors. For example, the proposed EU regulation [58] for new buildings requests 0 kWh/m^2^ yearly average energy use (Net Zero Energy Building [59], which is a very similar concept, to the one in the TWA scenario.

Even between the two alternative scenarios, there is a significant difference in the electricity use. The deviation between the TWA and CLEVER is explained by the fact that the target in the CLEVER is to increase the electricity use in Hungary to the European average, however, the TWA considers that this increase would push the sustainability limits and it is also not necessary from a quality-of-life perspective; in other words, it would be possible to increase well-being and, even reduce per capita use, through knowledge and awareness [60]. While the TWA uses the strong sustainability aspect [61], where environmental limits (and humanity's long-term interest in preventing ecological collapse) are at the heart of the issue, the CLEVER scenario emphasizes the social aspect even though environmental aspects are also part of the concept as described in the “Doughnut theory” [46]. In the NECPs, the focal point is economic development without the ambition to substantially reduce the environmental impacts.

Electrification as one of the most important elements of the energy transition can be observed in all four scenarios. Nevertheless, the electrification of key sectors will require increased electricity use. One example is the transportation sector where the ratio of BEV is 56 % in TWA and 61 % in the CLEVER by 2050. This step would result in a decrease in primary energy use by 75 % due to the much higher efficiency of electric engines and their use in practice (regenerative breaking). Structural reforms, such as teleworking [62], enhancing public transport [63], and demand-reducing transport strategy [64], could decrease energy demand significantly, even by 77 % [65]. There are equally significant opportunities to reduce energy use in buildings, including their electricity demand [59,66].

### 5.3 Electricity supply

Renewable sources are at the centre of both the CLEVER and TWA while nuclear fuel and solar PV are the key sources in the NECPs’ WAM scenario.

The NECPs show a significant increase by 2030 because of the launch of new nuclear reactors (2400 MWp), which would work in parallel with the older reactors. The supply is reduced by 2040 because of the decommissioning of the old reactors (2000 MWp). Consequently, the reliance on nuclear fuel increases energy dependence as the fuel is imported from Russia. Added to this are the gas imports associated with the new CCGT gas demand of 1650 MW according to the NECP 2023.

The lack of wind energy and underestimated role of biogas in the NECP 2019 WAM is a contrast to other studies and situations in other European countries, including neighbouring countries. Moreover, its distorted resource structure with a mix almost exclusively based on nuclear and solar PV supply reduces energy security.

### 5.4 Electricity imports, surplus generation, and self-sufficiency

From an environmental perspective, the share of renewables in electricity use should be considered when analysing imports and surplus generation. Despite the high theoretical surplus in the TWA, it should be noted that nearly 100 % of the electricity use would be from domestic renewable sources by 2050. In the CLEVER more than 70 % of the electricity use would be from domestic renewable sources by 2050. However, in the NECP 2019 WAM the share of renewable electricity would be nearly 30 % by 2040 and in the NECP 2023 WAM it would be 41 % by 2050. Therefore, a weakness of the NECP 2023 WAM is that the generation mix of this scenario results in a significant amount of surplus electricity without the benefit of meeting the demand with mostly renewable electricity.

Regarding electricity imports, the NECP 2023 WAM requires more imports than the TWA (after 2030) and fewer electricity imports than CLEVER and NECP 2019 WAM because of the significant nuclear capacity. However, this capacity will impact the theoretical surplus generation because the flexibility of this type of power station is limited.

The NECP 2019 WAM shows a relatively high import requirement by 2040 due to the phase-out of the old nuclear reactors. This scenario shows a low theoretical surplus electricity generation which is explained by the low share of renewables in the mix.

TWA and CLEVER show a high volume of theoretical surplus generation which is also explained by the high shares of wind and solar power. Import requirements by 2050 are the lowest in the TWA and the highest in the CLEVER. The reason for the lowest import requirements in the TWA is that this scenario proposes the most significant capacity of wind power among all scenarios. The reason for the high import requirements in the CLEVER is that this scenario is based on the cooperation between European countries and the imported electricity would be from renewable sources that would help balance the electricity grid at the continental level.

The self-sufficiency index shows that wind turbines, photovoltaics, and bioenergy could provide a significant portion of the required electricity. The choice of these resources would reduce the imports of nuclear fuel and natural gas for electricity generation.

It is suggested that self-sufficiency is achievable 79 % of an average week of January in the TWA, 52 % in the CLEVER, and 27 % in the NECP 2023 WAM scenario. Self-sufficiency levels are higher in January in those scenarios with more wind energy capacity (particularly TWA).

In the week of June, self-sufficiency is achievable in 95 % in the TWA, 77 % in the CLEVER, and 52 % in the NECP 2023 WAM scenario. Self-sufficiency levels are higher in all scenarios in June due to significant solar PV capacity, particularly in the CLEVER and NECP 2023 WAM scenario.

#### 5.4.1 Uncertainties associated with the model

One of the main limitations of the study is the lack of detail on the possible changes to the hourly electricity demand pattern due to the widespread use of technologies such as electric cars and heat pumps for heating and cooling. This detail would enhance the accuracy of the mismatch between supply and demand in the long-term.

While energy scenarios can support decision making, deterministic models come with a high level of uncertainty. Mirakyan and De Guio [67] pointed out that deterministic models often underestimate the impact of uncertainties since the outcome of those models is the same for a particular set of inputs, unlike stochastic models that predict outcomes that account for a certain level of unpredictability. Deterministic models focus on evaluating the impact of predefined energy systems while optimization models will endogenously suggest an optimal energy system according to a set of criteria.

The uncertainty of optimization models is connected to the optimization criteria (e.g., if the goal is to minimize system costs, the uncertainty of energy prices will influence the result). In a deterministic mode, each scenario assumption contains a level of uncertainty. These scenarios may not respond well to variations to input parameters such as energy use patterns, renewable energy supply (e.g., wind power generation), yearly costs and CO_2_ emissions [68]. Therefore, these uncertainties make the comparison between scenarios less reliable as they will affect the reliability of the system. Further analysis of uncertainties, particularly of input parameters, is recommended to avoid choosing a suboptimal development pathway.

## 6 Conclusion and recommendations

The paper presented a methodology to compare targets for the development of the electricity system from four different studies. The analysis was focused on indicators relevant for developing a more sustainable energy system. Based on the comparison, the targets set in the NECP were discussed.

The key finding of this paper is that the NECPs have modest targets for the transition to a more sustainable energy system. Based on the other scenarios, the final energy use could be 20 % lower. The second crucial policy-related target is the import dependency which could be reduced along with reducing the use of nuclear electricity. The reason is that nuclear energy in the country is heavily dependent on imported fuels, increasing the vulnerability of Hungary. The third main target under consideration is the share of renewable energy technologies that are capable of a truly sustainable supply. According to the alternative scenarios (TWA and CLEVER), the potential for renewable electricity utilization is significant in Hungary, particularly wind energy.

Based on the comparison, the following conclusions and observations are proposed.

• The CLEVER scenario is the one with a focus on social aspects and the potential to reduce energy use based on behavioural change.
• The NECPs do not consider the challenges of social and environmental sustainability and the opportunities for sufficiency and efficiency.
• The TWA is the most ambitious in decreasing primary energy use. This is the oldest of the four scenarios which shows that the concept of sufficiency is not new, but it was still neglected in the NECP scenarios.
• The NECPs failed to consider the fast improvement in solar PV and wind technologies.
• The estimated surplus electricity supply is significant in all the scenarios; the difference is the share of renewable electricity use which is lower in the NECPs.
• The NECP need to be rethought to create more ambitious targets using multidisciplinary approaches and methods, where the human aspect has a more significant role, and where development can happen without increasing energy use and environmental burden.
• One of the stronger points of the CLEVER scenario is the cooperation between neighbouring countries to cope with challenges in supply and reduce the dependency on one single country. The CLEVER as a scenario with intermediary targets could be a starting point for rethinking the importance of sufficiency as well as international cooperation - NECPs should widen their national focus to an EU-level approach, adding a chapter on international cooperation (electricity and fuel import/export).

The NECP of Hungary proposes a slow and unclear path for transitioning to a sustainable energy system. Recent events such as the government launching a coal program that subsidizes coal mining in Borsod-Abaúj-Zemplén county reinforce that the transition lacks clear direction [[69], [70], [71]]. A first step to revise the NECP could be a broad public discussion informed by scientific arguments. To advance energy policy, Hungary should support dedicated research on energy and climate planning, involving experts from various fields, e.g., social science, education, resource management, etc. The government should lead this effort to produce rigorous alternative energy scenarios, ideally in collaboration with diverse scientific workshops and think tanks.

## CRediT authorship contribution statement

**Béla Munkácsy:** Writing – review & editing, Writing – original draft, Supervision, Methodology, Conceptualization. **Csaba Csontos:** Writing – review & editing, Resources. **Ádám Harmat:** Writing – original draft, Resources. **José Campos:** Writing – review & editing, Writing – original draft, Visualization, Software.

## Data availability statement

Data associated with this study has not been deposited into a publicly available repository but will be made available on request.

## Declaration of competing interest

The authors declare that they have no known competing financial interests or personal relationships that could have appeared to influence the work reported in this paper.
